# Synchronizing Allelic Effects of Opposing Quantitative Trait Loci Confirmed a Major Epistatic Interaction Affecting Acute Lung Injury Survival in Mice

**DOI:** 10.1371/journal.pone.0038177

**Published:** 2012-05-29

**Authors:** Daniel R. Prows, William J. Gibbons, Benjamin B. Burzynski

**Affiliations:** 1 Department of Pediatrics, University of Cincinnati College of Medicine, Cincinnati, Ohio, United States of America; 2 Division of Human Genetics, Cincinnati Children's Hospital Medical Center, Cincinnati, Ohio, United States of America; Johns Hopkins School of Medicine, United States of America

## Abstract

Increased oxygen (O_2_) levels help manage severely injured patients, but too much for too long can cause acute lung injury (ALI), acute respiratory distress syndrome (ARDS) and even death. In fact, continuous hyperoxia has become a prototype in rodents to mimic salient clinical and pathological characteristics of ALI/ARDS. To identify genes affecting hyperoxia-induced ALI (HALI), we previously established a mouse model of differential susceptibility. Genetic analysis of backcross and F_2_ populations derived from sensitive (C57BL/6J; B) and resistant (129X1/SvJ; X1) inbred strains identified five quantitative trait loci (QTLs; *Shali1-5*) linked to HALI survival time. Interestingly, analysis of these recombinant populations supported opposite within-strain effects on survival for the two major-effect QTLs. Whereas *Shali1* alleles imparted the expected survival time effects (*i.e.*, X1 alleles increased HALI resistance and B alleles increased sensitivity), the allelic effects of *Shali2* were reversed (*i.e.*, X1 alleles increased HALI sensitivity and B alleles increased resistance). For *in vivo* validation of these inverse allelic effects, we constructed reciprocal congenic lines to synchronize the sensitivity or resistance alleles of *Shali1* and *Shali2* within the same strain. Specifically, B-derived *Shali1* or *Shali2* QTL regions were transferred to X1 mice and X1-derived QTL segments were transferred to B mice. Our previous QTL results predicted that substituting *Shali1* B alleles onto the resistant X1 background would add sensitivity. Surprisingly, not only were these mice more sensitive than the resistant X1 strain, they were more sensitive than the sensitive B strain. In stark contrast, substituting the *Shali2* interval from the sensitive B strain onto the X1 background markedly increased the survival time. Reciprocal congenic lines confirmed the opposing allelic effects of *Shali1* and *Shali2* on HALI survival time and provide unique models to identify their respective quantitative trait genes and to critically assess the apparent bidirectional epistatic interactions between these major-effect loci.

## Introduction

Acute lung injury (ALI) and acute respiratory distress syndrome (ARDS) continue to have high mortality [1]–[4], despite decades of research and numerous randomized clinical trials [5]–[10]. A significant reduction in mortality has been achieved with protective ventilation strategies [11], but pharmacological attempts remain disappointing [6], [10], [12]. Among the supportive measures available, use of supranormal oxygen (O_2_) to correct the severe hypoxemia is integral to the management of ALI/ARDS patients. Oxygen therapy is also an essential treatment in many other acute (*e.g.*, trauma) and chronic (*e.g.*, COPD) cases. Nonetheless, the paradoxical detrimental pulmonary effects from high O_2_ levels remain a complicating factor in critical care medicine. Basic research, on the other hand, has taken advantage of the damaging oxidative capacity of high-dose O_2_ in laboratory animals to directly study ALI development and pathology [13]–[18], and to identify and critically evaluate potential protective and therapeutic strategies [19]–[22].

The biologic and pathologic features of ALI are well established [4], [23]; yet, the identification of key genes affecting differential susceptibility and outcome has been elusive. With a long term goal to determine the critical genes controlling oxidant-induced ALI morbidity and mortality, we have established a susceptibility mouse model using hyperoxic acute lung injury (HALI) survival time for genetic analysis and gene discovery [24]. Two distinct large recombinant populations were generated for quantitative trait locus (QTL) analysis, using sensitive C57BL/6J (B) and the considerably more resistant 129X1/SvJ (X1) inbred mouse strains as the progenitors. Analysis of F_2_ mice identified 5 QTLs (designated *Shali* for ***S***urvival time with ***h***yperoxic ***a***cute ***l***ung ***i***njury) significantly linked to HALI survival time on Chr 1 (*Shali1* and the male-specific QTL, *Shali5*), Chr 4 (*Shali2*), Chr 9 (*Shali4*), and Chr 15 (*Shali3*) [25]. The large backcross population also identified *Shali1*, *Shali2*, and *Shali3* and, along with the F_2_ data, consistently suggested that the *Shali1* locus on Chr 1 and the *Shali2* locus on Chr 4 had opposing allelic effects on overall HALI survival time within each inbred strain [26]. Specifically, QTL analysis of recombinants derived from the X1 and B progenitor strains determined that *Shali1* directly correlated with the overall survival time trait of the parental strains, with resistant X1 strain alleles leading to an increased mean HALI survival time and sensitive B strain alleles yielding increased sensitivity. Conversely, phenotype data of backcross and F_2_ recombinants supported that *Shali2* had allelic effects in opposition to *Shali1*, with sensitive strain B alleles for *Shali2* prolonging HALI survival time and resistant strain X1 alleles for *Shali2* increasing sensitivity. The individual contributions of these QTLs to the overall survival time were estimated to change the survival time about 23 and 15 hrs in the corresponding direction for *Shali1* and *Shali2*, respectively [25].

Besides the opposing allelic effects on overall HALI survival time for the two main QTLs, three additional factors have added significantly to the complexities of these genetic studies. First, the sensitive B strain showed a significant sex difference, with males more resistant than females. Interestingly, although no linkage was mapped to the X-chromosome, we did identify a male-specific QTL (*Shali5*) on distal Chr 1 [25]. Second, even though the X1 strain is considered a resistant strain (based on mean survival time; MST), the penetrance (*i.e.*, the proportion of mice carrying the QTL and demonstrating the phenotype) of the resistance trait is low, with a total male-female average of ∼33% penetrance. And, among the one-third of X1 mice that survived to the resistance threshold (>153 hrs), only about a third of these (*i.e.*, ∼10–15% of total X1 mice) reached a survival time of 200 hrs. This decreased penetrance can significantly increase the difficulties of identifying the resistance gene(s). Third, at least one additional locus (*i.e.*, *Shali5* and possibly *Shali3*) also showed an allelic effect on HALI survival opposite to that expected, with B alleles predicted to confer added HALI resistance and X1 alleles increased sensitivity. These complexities are likely inherent to most complex traits [27]–[32].

To verify the predicted QTL effects of the major loci *in vivo*, reciprocal congenic lines were constructed for *Shali1* and for *Shali2* on both parental strain backgrounds, which involved substituting the respective donor B strain segment onto the resistant X1 strain and substituting the donor X1 strain segment onto the sensitive B strain for both QTLs. Effects of this genetic restructuring on trait penetrance was also examined. Consistent with our earlier QTL analyses results, these congenic lines demonstrated significant changes in HALI survival time compared to their corresponding progenitor strain, thereby providing strong *in vivo* evidence for the existence and capture of these QTLs. By generating a congenic line on the resistant X1 strain background that was even more sensitive than the sensitive B strain, we further validated the bidirectional alleles of these QTLs. And, by significantly augmenting the overall effects on HALI survival time in both directions, these congenic lines revealed important epistatic interactions between the two *Shali* loci. These data support that the validated congenics represent an excellent model system to further delineate these loci and can be expanded to include congenics for additional putative *Shali* loci and other critical genes affecting this multigenic response.

## Materials and Methods

### Mice

C57BL/6J (B) mice (*i.e.*, the HALI-sensitive strain) and 129X1/SvJ (X1) mice (*i.e.*, the HALI-resistant strain) were purchased from the Jackson Laboratory (Bar Harbor, ME) and used in the construction of the reciprocal X1.B (*i.e.*, X1 strain background with a B-derived QTL substitution) and B.X1 (*i.e.*, B strain background with a X1-derived QTL substitution) congenic lines for *Shali1* and *Shali2*, on chromosomes 1 and 4, respectively. Note: to avoid confusion, the “X1” abbreviation is used in this report (and all future communications) to denote the resistant inbred strain, which differs from our previous published work using “S” as the abbreviation for the resistant strain. B strain mice were brother-sister mated in-house to generate breeders and sensitive-strain controls for exposures. Inbred stocks were replaced at least every 10 generations to reduce strain drift. Due to their high rate of conjunctivitis, X1 mice were purchased (Jackson Laboratory) as needed for use as breeders and as resistant-strain controls for the hyperoxia exposures.

### Ethics Statement

Mice were handled in accordance with the protocols approved by the IACUC of Cincinnati Children's Hospital Medical Center.

### QTL genotype analysis

To demonstrate the reciprocity of the different allele combinations at the representative QTL peak loci and to help quantify the opposing effects of *Shali1* and *Shali2* in the model, our previous QTL dataset of 840 F_2_ recombinants [25] was re-sorted and re-plotted for direct allelic comparisons. All F_2_ mice (n=840) were typed for *D1Mit303* (the peak marker of *Shali1*) and sorted into three genotypes groups: those homozygous for X1-derived alleles, heterozygous, or homozygous for B-derived alleles; the MST was calculated for each allelic group of mice. Similarly, the 837 F_2_ mice successfully typed for *D4Mit308* (the peak marker of *Shali2*) were segregated into the three genotypic groups and their MSTs calculated. Next, the combined genetic contribution for *Shali1* and *Shali2* was estimated by first sorting the 837 mice typed for both *D1Mit303* and *D4Mit308* markers into the nine different possible genotypes, and then calculating and plotting the MSTs for these nine groups of mice.

### Construction of consomic and congenic lines

The consomic and congenic lines described in these studies were generated over the past 3–4 years. Because Chr 1 contained two distinct QTLs separated by a large distance (*i.e.*, *Shali1* and the male-specific QTL, *Shali5*), and Chr 4 maintained a high LOD score for a large portion of the chromosome [25], we initiated construction of consomic lines for these two chromosomes. Reciprocal lines for *Shali1* and *Shali2* are designated herein as B.X1-1 and X1.B-1 and B.X1-4 and X1.B-4, respectively; background strain is listed first, followed by the substituted chromosome strain and chromosome number. A general backcross strategy and marker-assisted selection protocol [33], [34] was employed, using donor males and recipient X1 strain or B strain females, where indicated. Male donors for breeding in the next generation were selected to be heterozygous for the entire donor chromosome of interest, and carrying the most recipient strain homozygosity for the remainder of the genome. Directional backcrosses were performed to ensure the corresponding recipient strain-derived mitochondria and Y-Chr in the final lines. A genomewide SNP analysis (Mouse MD Linkage Panel; Illumina, San Diego, CA) of lines at N_3–4_ was performed at Cincinnati Children's Genetic Variation and Gene Discovery Core (http://dna.chmcc.org/), and additional microsatellite markers were typed to directly target removal of the remaining heterozygous regions.

Near the end stages of the multi-generation screening process of constructing the four reciprocal consomic strains, select recombinants were identified that contained or did not contain a conservative *Shali1* or *Shali2* interval. After reproducing each recombination in the opposite sex by backcrossing to the appropriate parental strain, the congenic regions were fixed to homozygosity to 1) confirm the QTL effect and determine the level of overall phenotype represented by the captured region, 2) further resolve the congenic intervals, and 3) maintain the lines. The Mouse Universal Genotyping Array (MUGA; GeneSeek, Lincoln, NE), which includes >7,800 SNPs at an average spacing of 325 Kb, was used to verify the genetic makeup of the most relevant final consomic and congenic lines. Small regions of heterozygosity still remaining at this stage were eliminated with additional backcrosses using targeted microsatellite or SNP markers and the final lines reestablished to homozygosity.

### DNA preparation

Genomic DNA was isolated from 2–3 mm tail clips using a commercial DNA extraction kit (Wizard Genomic DNA, Promega; Madison, WI). A Nanodrop 8000 (Thermo Fisher Scientific; Waltham MA) was used to analyze samples for purity (A_260_/A_280_) and DNA content (A_260_). DNAs were diluted to ∼20 ng/µl for PCR requiring agarose gel separation or to ∼5 ng/µl for PCR using fluorescence genotyping.

### Genotype analysis

Primer pairs for polymorphic markers between the B and X1 strains were purchased from IDT (Coralville, IA). PCR was performed in 15-µL volumes in 96-well plates (USA Scientific; Ocala, FL) using a 4-block thermocycler (BioRad, Model PTC-225 or PTC-240) as described previously [25]. Markers with PCR product allele sizes of 5% or more were separated by 2.5–4% agarose (ISC BioExpress; Kaysville, UT) gels and stained with ethidium bromide. Microsatellite markers <5% different in allele size were amplified from 20 ng total DNA using fluorescent primers synthesized by Applied Biosystems (ABI; Foster City, CA) and protocols provided. Fluorescent PCR products were separated using an ABI-3730*xL* sequencer located at the Cincinnati Children's Genetic Variation and Gene Discovery Core (http://dna.chmcc.org/) and genotypes ascertained using GeneMapper software (V3.5, ABI). Besides using targeted microsatellite analysis whenever possible, real-time qPCR of individual SNPs (ABI) was used to remove any remaining small heterozygous regions identified by the MUGA SNP panel (GeneSeek, Inc.).

### Hyperoxia Exposures

Mice in standard shoebox cages with food and water *ad libitum* were placed inside a 0.13-m^3^ Plexiglas inhalation chamber (manufactured to specifications by Stellar Plastics, Detroit, MI) and exposed continuously to >95% O_2_ until death. Each chamber housed up to 9 cages with up to 4 mice per cage; individual mice were clearly visible from above and through the sides of cages. O_2_ level in the chamber was continuously controlled using a ProOx 110 portable O_2_ monitor (Biospherix, Redfield, NY). Status of the exposures was closely monitored, such that the time between measures was within 5% error of the overall exposure to that point. The exposure time used for each mouse was the mean time between the last time check and the time the mouse was identified as dead. Mice were exposed between 6 and 12 weeks of age and, to prevent hypoxic seizure upon opening the chamber, each exposure was continued uninterrupted until all mice within the chamber succumbed. Because of the large number of control mice exposed over the years, and the decreased penetrance seen in the resistant X1 inbred strain, the B and X1 strains are presented as historic controls for group comparisons, and include data previously published [24] combined with new data for the total control mice.

### Statistical analyses

Results are presented as mean survival times (MSTs) ± SEM. Survival data related to the QTL genotype analyses and the strain comparisons were not normally distributed. For QTL genotype analyses, one-way ANOVA on ranks was used to compare MSTs of F_2_ mice with the same single-marker (*D1Mit303* or *D4Mit308*) or two-marker (*D1Mit303*+*D4Mit308*) genotypes (*i.e.*, B-B, H, or X1-X1). Tukey *posthoc* adjustments for multiple comparisons were used to keep the overall family-wise type I error rate at 0.05. A Mann-Whitney-Wilcoxon (MWW) U-test was used for strain comparisons. Statistical differences were first assessed for sex in the control B and X1 strains. X1 strain males and females did not differ (p=0.72), whereas B strain males were significantly more resistant than B strain females (p=7.9×10^−5^). Therefore, all data were stratified by sex. Sex-specific between group comparisons (B *vs.* each B.X1-1 congenic, B *vs.* each B.X1-4 congenic, X1 *vs.* each X1.B-1 congenic and X1 *vs.* each X1.B-4 congenic) were assessed using a MWW U-test and a Bonferroni correction (38 tests). A sex-matched comparison of the hypersensitive X1.B-1A line to the sensitive B strain control was also performed (MWW U-test). After accounting for multiple comparisons, differences were considered statistically significant when *p*<0.00132 (*i.e.*, *p*<0.05/38).

## Results

### Allelic analysis of *Shali1* and *Shali2*

**Figure 1:**
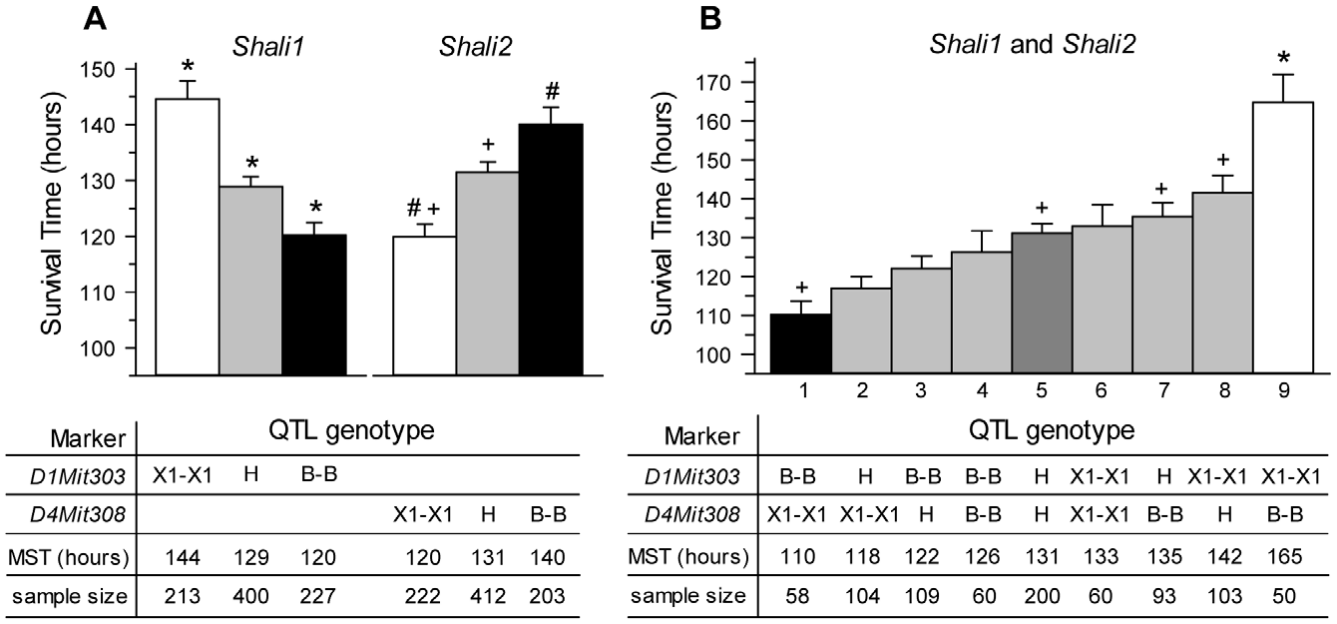
Mean survival times of mice grouped by genotypes at markers with peak LOD scores for *Shali1* and *Shali2*. The F_2_ population (n=840) generated from sensitive B and resistant X1 mice [25] was sorted into groups based on their genotypes (X1-X1, H, or B-B) at the peak MIT marker representing *Shali1* (*D1Mit303*; 1–303) or *Shali2* (*D4Mit308*; 4–308) (panel A) or at both peak markers (panel B). Survival times of mice for each genotype group was determined and plotted as the mean (MST) ± SEM. Groups that differed from each other are marked with the same symbol. Panel A shows a near mirror image of the genotype bar plots, indicative of the reciprocal allelic effects of *Shali1* and *Shali2*. For *Shali1* (*D1Mit303*), F_2_ mice for all three genotype groups differed from each other (indicated by *). For *Shali2* (*D4Mit308*), F_2_ mice homozygous for X1 alleles differed from mice H or B-B (+), and mice B-B differed from those X1-X1 (#). Panel B shows a gradation of effect for the 9 different pairwise genotypes, with the polar extremes for MSTs (*i.e.*, bars 1 *vs.* 9) occurring with the B-B, X1-X1 (high sensitivity) and X1-X1, B-B (high resistance) reciprocal genotypes at *Shali1* and *Shali2*, respectively. Moving inward from each end gives group comparisons for each reciprocal genotype pair (*i.e.*, bars 2 *vs.* 8, bars 3 *vs.* 7 and bars 4 *vs.* 6). The group of mice heterozygous for both markers fell in the center (bar 5), with a MST intermediate to all other groups. n, represents the number of F_2_ mice successfully genotyped for each (or both) MIT marker(s). F_2_ mice with genotype 9 (*i.e.*, X1-X1, B-B at *D1Mit303* and *D4Mit308*, respectively) were the most resistant and differed from all other genotypes (indicated by *). F_2_ mice with the reciprocal B-B, X1-X1 genotype were the most sensitive and, besides differing from genotype 9, also differed from genotypes 5, 7, and 8 (+).

Our previous QTL data suggested that the *Shali1* and *Shali2* allelic effects opposed each other in HALI survival time [24]–[26]. To quantitate and compare these QTL effects on survival time, allelic analysis of the major QTLs was performed in the F_2_ dataset [25]. For this, the allelic effects of the peak markers for *Shali1* and *Shali2* loci were calculated, first for the separate QTLs and then for both QTLs. F_2_ mice with homozygous X1 alleles for *D1Mit303* were the most HALI resistant, with heterozygotes intermediate in MST and mice homozygous for B alleles at *D1Mit303* being most sensitive. This directly correlated with X1 being the resistant strain. In contrast, F_2_ mice that were homozygous for B alleles at *D4Mit308* were the most HALI resistant, even though the inbred B strain is HALI sensitive. Similarly, F_2_ mice that were homozygous for X1 alleles at *D4Mit308* were most sensitive, even though inbred X1 mice are a resistant strain. Mice heterozygous at *D4Mit308* were again intermediate in survival time. Because this data provided a key finding that formed the basis of the breeding strategies reported in this study, we have re-plotted these data to clearly demonstrate this inverse relationship. In this format, the opposing actions of the homozygous B and X1 alleles for *Shali1* and *Shali2* can be seen as near mirror images, showing a similar, but opposite effect of the two QTLs and HALI survival times (Figure 1A). The overall survival time with homozygous X1 alleles at *D1Mit303* (145 hrs) was similar to that for homozygous B alleles at *D4Mit308* (140 hrs). Heterozygotes for either QTL marker also had similar MSTs to each other (both ∼130 hrs) and to the control X1 strain (133 hrs).

The genotype and phenotype data for the F_2_ population was further re-sorted into groups of mice that carried one of the 9 possible pairwise genotypes at *D1Mit303* and *D4Mit308*; data for the nine groups was displayed according to increasing MST (Figure 1B). A visual comparison of these 9 groups revealed a consistent pattern for the reciprocal allelic pairs. For example, bars 1 and 9, which include the reciprocal (B-B, X1-X1) and (X1-X1, B-B) genotype pair for the *Shali1* and *Shali2* markers, respectively, demonstrated the polar extremes of MSTs among the 9 genotype pairs. Moving inward from each end in Figure 1B, we see that bars 2 and 8, 3 and 7, and 4 and 6 directly matched reciprocal pairs at the corresponding increasing and decreasing MSTs. The group of mice heterozygous (*i.e.*, B-X1 or X1-B; listed as H) for both *Shali* markers was in the exact center of the range, as would be predicted by the reciprocal effects. Again, the group of mice that were heterozygous at both loci had a MST no different than the control X1 strain.

**Figure 2:**
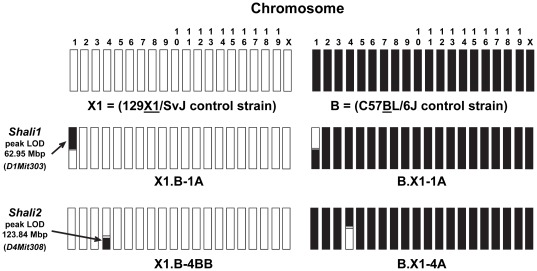
Genomic makeup of the *Shali1* (Chr 1) and *Shali2* (Chr 4) reciprocal congenic lines. A schematic overview comparing the 19 autosomes and X chromosome for the X1 (white) and B (black) control inbred strains along with the major *Shali1* and *Shali2* reciprocal congenic strains generated on the recipient X1 strain with B strain donor (X1.B-1A and X1.B-4BB) or recipient B strain with X1 strain donor (B.X1-1A and X1.B-4A). Small regions of unknown parental origin, which map between known SNP markers, are colored grey. MIT markers representing the peak linkage (LOD) score for the *Shali1* and *Shali2* QTLs are shown at the left, with approximate map location relative to chromosome and transferred region.

To validate these *in vitro* allelic predictions *in vivo*, we generated a pair of reciprocal congenic lines for these two QTLs on both parental background strains. Figure 2 displays a genomic overview of the most important reciprocal congenic strains for *Shali1* (X1.B-1A and B.X1-1A) and *Shali2* (X1.B-4BB and B.X1-4A) compared to each other and to the X1 and B inbred progenitor strains. Specific details of these lines are described below. From this illustration, one can quickly note the genomic differences between these congenic lines and their respective progenitor (recipient) strains.

### Generation of *Shali1* lines

#### Reciprocal consomics

**Figure 3:**
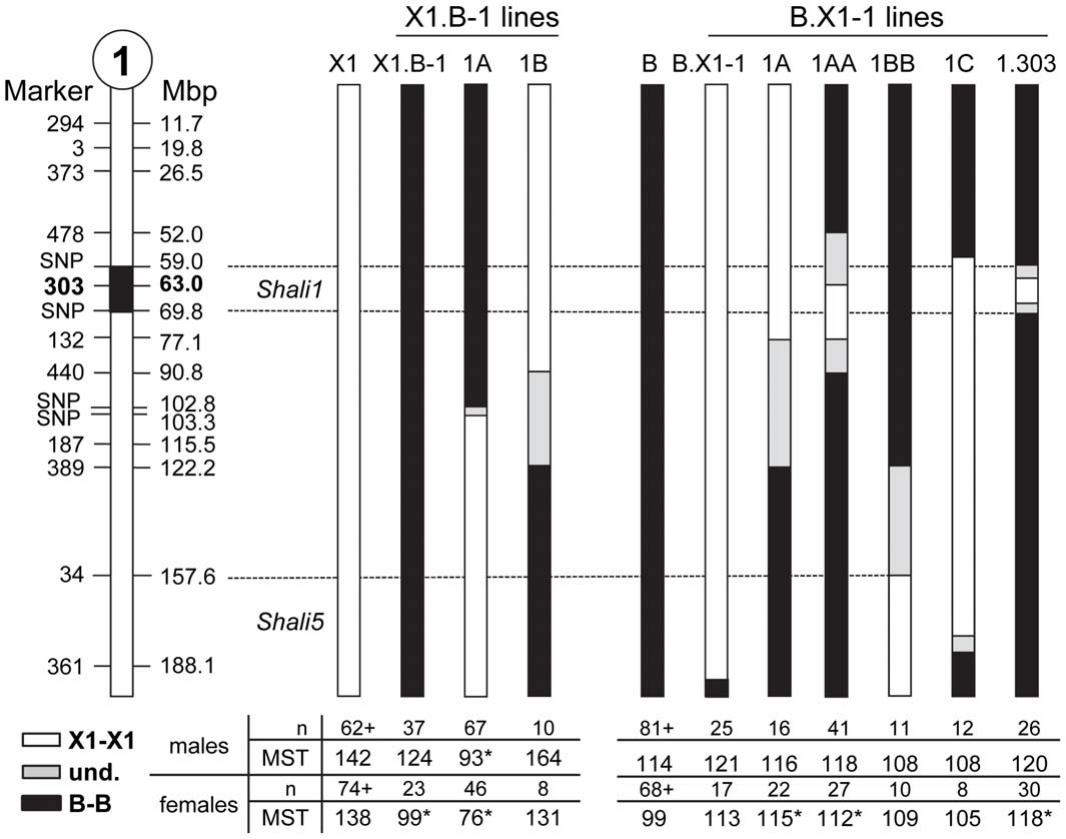
Schematic summary of the reciprocal X1.B-1 and B.X1-1 consomic, congenic and subcongenic lines for *Shali1*. Data represent chromosome 1 of each strain, with X1 (solid white bar) and B (solid black bar) representing the recipient background strain of X1.B-1 and B.X1-1 lines, respectively. Chromosome 1 on the far left depicts MIT markers and positions (Mbp), along with the putative *Shali1* interval (black box). The current validated *Shali1* and *Shali5* intervals are indicated by dashed lines. X1.B-1 and B.X1-1 represent reciprocal consomic lines (full chromosome substitution). For X1-B-1 lines (left half), regions in black denote the substituted chromosome region, including the congenic lines X1.B-1A and X1.B-1B. For B.X1-1 lines (right half), regions in white represent the substituted regions. B.X1-1A and B.X1-1C are congenic lines and B.X1-1AA and B.X1-1BB are subcongenic lines of B.X1-1A and B.X1-1B (died), respectively. B.X1-1.303 was derived from a rare double recombinant identified early the screening process and fixed after removing all donor regions outside of the ∼11-Mbp area around marker *D1Mit303*. Grey regions represent areas not yet tested for parental origin. Tables below each set of congenics summarizes the mean survival time (MST) and sample size (n) for males and females of each line. *significantly different than same-sex background strain (*p*<0.00132; MWW U-test with Bonferroni correction for 38 group comparisons). +, represents a historical control and includes combined data for new and previously reported controls [24], [25].

Previous QTL results supported an increased survival time for X1 alleles and increased sensitivity for B alleles occurring at the *Shali1* locus. QTL results also suggested that X1 and B alleles for *Shali5*, a putative male-specific QTL on distal Chr 1, had reversed effects on HALI survival time in males when compared to *Shali1* (*i.e.*, X1 alleles caused increased sensitivity in males, whereas B alleles added resistance in males). Reciprocal Chr 1 consomic lines were constructed to capture both *Shali1* and *Shali5* QTLs in the same background strain and to serve as the source and backup lines for current and future Chr 1 congenics (Figure 3). The B.X1 and X1.B reciprocal consomic lines for *Shali1* were fixed to homozygosity using microsatellite markers and/or the Illumina medium-density SNP array. The final genetic makeup of the reciprocal Chr 1 consomics was determined using the newly available MUGA SNP panel (GeneSeek, Inc.). The X1 alleles for the distal ∼5 Mbp of Chr 1 were not captured in the B.X1-1 consomic line.

#### Reciprocal congenics

The *Shali1* congenic strains were conservatively constructed to contain more than the 95% confidence interval transferred from the B strain onto the resistant X1 strain background (designated herein as X1.B-1A), or the reciprocal congenic line that contained *Shali1* from the X1 strain, transferred to the sensitive B strain background (designated herein as B.X1-1A) (Figure 3). The X1.B-1A congenic strain originated from a recombinant male that was heterozygous for the proximal half of Chr 1, but homozygous for X1 alleles at the tested microsatellites throughout the remainder of the genome. A female with the same recombination was generated from, and then bred with, the original male to fix the X1.B-1A line. Final MUGA SNP analysis of the fixed X1.B-1A congenic line mapped the crossover between SNPs at 102.558 Mbp and 103.336 Mbp. Similarly, a recombinant male carrying the distal half of Chr1 (and *Shali5*), with a crossover between *D1Mit440* and *D1Mit389* was also identified and fixed to homozygosity to capture the putative male-specific QTL (Figure 3).

B.X1-1 congenics and subcongenics were generated in much the same fashion. Three Chr 1 recombinants were identified and fixed to homozygosity (B.X1-1A, B.X1-1B, and B.X1-1C). The subcongenic line B.X1-1AA was generated from a B.X1-1A recombinant. The B.X1-1B line was lost shortly after the first litter; however, a recombinant identified from this litter led to the B.X1-1BB subcongenic line, which was fixed and maintained long enough to phenotype some offspring. The B.X1-1C line contained the *Shali5* region on distal Chr 1 and used to assess the male-specific effects of this QTL on overall survival time when placed on the B strain background. In addition, a congenic line (named B.X1-1.303), was generated from a rare double recombinant carrying the microsatellite marker *D1Mit303* and fixed to homozygosity. MUGA SNP analysis located the B.X1-1.303 *Shali1* interval to 59.0–69.8 Mbp (Figure 3).

### Generation of *Shali2* lines

**Figure 4:**
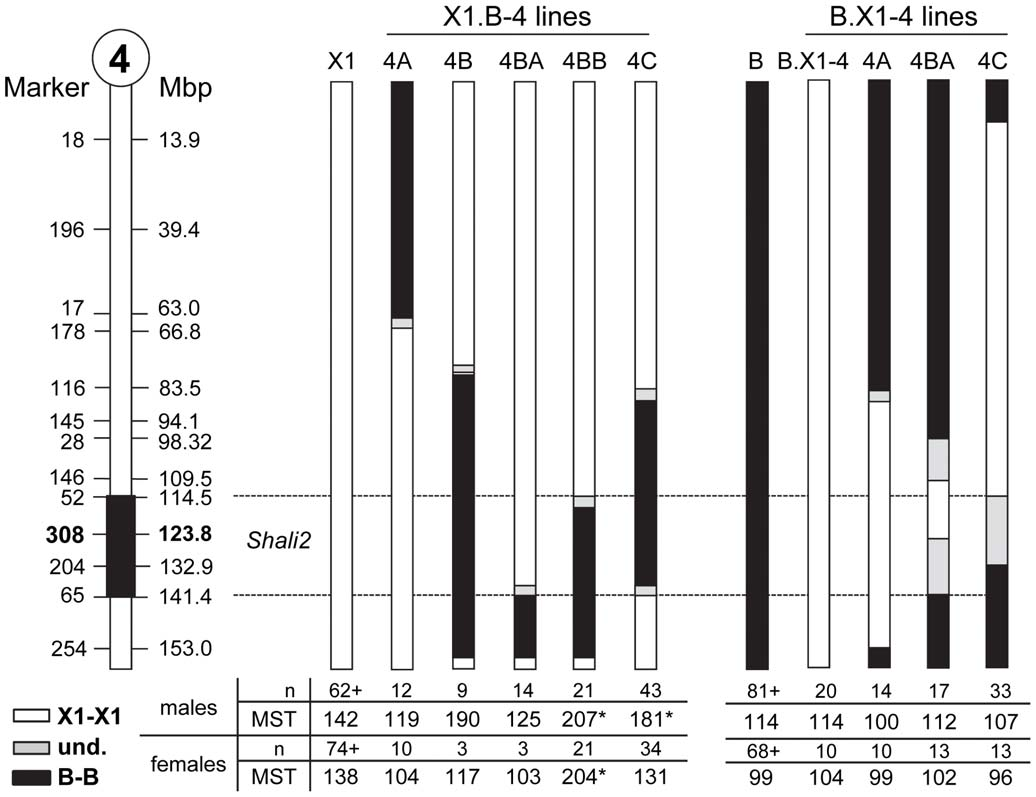
Schematic summary of B.X1-4 consomic and reciprocal X1.B-4 and B.X1-4 congenics and subcongenics for *Shali2*. Data represent chromosome 4 of each strain, with X1 (solid white bar) and B (solid black bar) representing the recipient background strain of X1.B-4 and B.X1-4 lines, respectively. Chromosome 4 on the far left depicts MIT markers and their genomic positions (Mbp), along with the *Shali2* interval (black box). The current *Shali2* interval is also indicated by dashed lines. Crossovers not matching dashed lines indicate SNPs from the Mouse Universal Genotyping Analysis (MUGA; GeneSeek) panel. For X1.B-4 lines (left half), regions in black denote substituted genome, with congenic lines X1.B-4A, X1.B-4B and X1.B-4C. X1.B-4BA and X1.B-4BB are subcongenic lines derived from the X1.B-4B line. For B.X1-4 lines (right half), regions in white represent substituted genome. B.X1-4 represents a consomic line (full chromosome substitution). B.X1-4A and B.X1-4C are congenic lines and B.X1-4BA is a subcongenic line of B.X1-4B (died). Grey regions represent areas not yet tested for parental origin. Tables below each set of congenic lines summarize mean survival time (MST) and sample size (n) for males and females of each line. *significantly different than same-sex background strain (*p*<0.00132; MWW U-test with Bonferroni correction for 38 group comparisons). +, represents a historical control and includes combined data for new and previously reported controls [24], [25].

The B strain is sensitive to the detrimental effects of hyperoxia. However, our earlier QTL results supported that F_2_ mice carrying homozygous B alleles for *Shali2* had increased resistance and those carrying homozygous X1 alleles from the resistant X1 strain demonstrated increased HALI sensitivity. Three overlapping congenic lines (X1.B-4A, X1.B-4B, and X1.B-4C) for Chr 4 were successfully produced from a short-lived Chr 4 consomic line (Figure 4). Collectively, these 3 congenics covered most of Chr 4; MUGA SNP analyses of these lines indicated that B-derived alleles for ∼66–81 Mbp were not captured. Due to poor breeding performance and poor mothering skills, few mice were generated from the X1.B-4A and X1.B-4B congenic lines. Fortunately, the results from exposing small numbers of mice for these lines quickly steered the breeding towards the X1.B-4B line, leading to the generation of X1.B-4BA and X1.B-4BB subcongenic strains (Figure 4). The X1.B-4BB line bred reasonably well, but the X1.B-4C line, which also contains the entire *Shali1* region, bred considerably better than all other Chr 4 lines, allowing us to more quickly test a larger population of mice for this line. The reciprocal B.X1-4 consomic line was bred to completion and recombinants were identified to establish three B.X1-4 congenics (B.X1-4A, B.X1-4BA, and B.X1-4C). All consomic and congenic lines were fixed to apparent homozygosity and exposed continuously to >95% O_2_. MSTs were determined for both sexes of each line and compared to the opposite sex and their appropriate parental controls.

### Exposures of the reciprocal consomics and congenics

#### *Chr 1: Shali1* and *Shali5* (Figure 3)

Based on results from the early Illumina SNP panel and targeted microsatellite analysis, the X1.B-1 consomic was fixed; therefore we began collecting survival time data. Recent MUGA SNP analysis of the X1.B-1 consomic identified 3 small regions still segregating for B alleles; however, we found no difference in HALI survival times for X1.B-1 mice still segregating for any of these 3 regions. Exposure of X1.B-1 mice demonstrated that B-derived full Chr 1 substitution yielded a slight, but not significant, overall decrease in survival time in X1.B-1 males compared to X1 males (124 hrs *vs.* 142 hrs; *p*=0.13). However, a significant increase in sensitivity was found for X1.B-1 females, as compared to X1 females (99 hrs vs. 138 hrs; *p*=3.1×10^−5^). In fact, the MST of the X1.B-1 females matched the MST of sensitive B strain females (99 hrs), and directly correlated with substitution of Chr 1 with the B strain.

**Figure 5:**
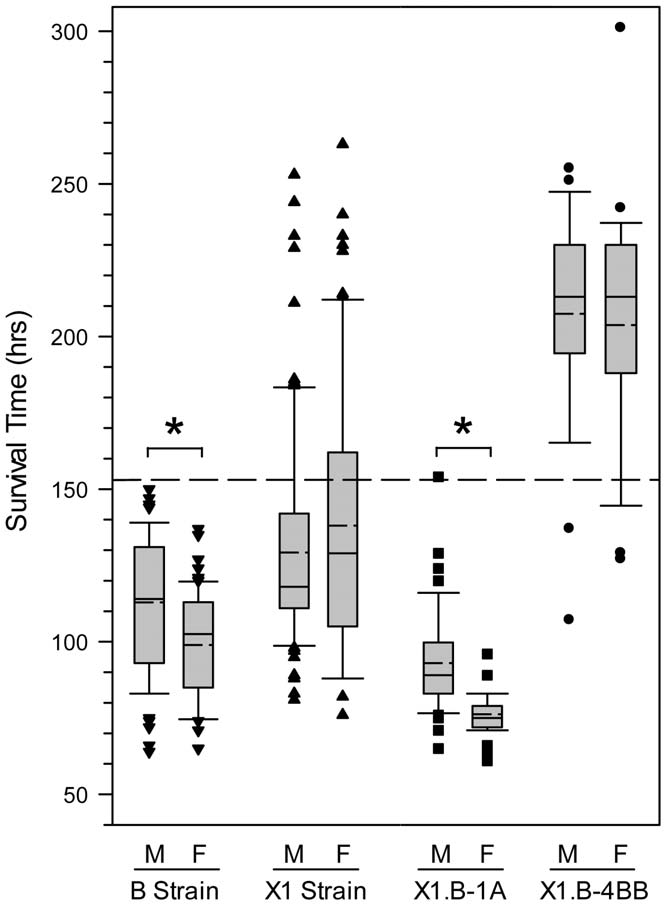
Box-plots comparing survival times for controls and the major *Shali1* and *Shali2* congenic lines. Boxes represent the 25^th^ to 75^th^ percentiles, and horizontal lines within the box represent median survival times; short dashed lines within boxes denote mean survival times. Whiskers (error bars) above and below the boxes indicate the 90^th^ and 10^th^ percentiles, respectively. The dashed line across the graph denotes the threshold survival time (153 hrs) for designation of the resistant phenotype [24]. Both sexes of all lines (B, X1.B-1A, and X1.B-4BB) differed significantly from the X1 progenitor strain. *, within-strain mean survival times of males (M) and females (F) differed significantly (*p*<0.00132).

A different picture emerged for the X1.B-1A congenic line, which has the resistant X1 strain background and substitution of the proximal half of Chr 1 from the sensitive B strain, including the *Shali1* interval (Figures 2 and 3). Therefore, compared to the X1.B-1 consomic strain, X1.B-1A mice lack B alleles for the distal half of Chr 1, which contains the *Shali5* region. As predicted, X1.B-1A mice were significantly more sensitive than X1 mice (males, *p*=3.6×10^−12^; females, *p*=2.2×10^−16^). Surprisingly, the X1.B-1A line also differed markedly in HALI sensitivity from the sensitive B strain control for males (*p*=7.2×10^−8^) and females (*p*=9.9×10^−11^). Similar to that found previously for the control B strain [24] and the X1.B-1 consomic line (described above), X1.B-1A males and females differed significantly (*p*=5.4×10^−10^) in HALI MSTs (Figures 3 and 5). X1.B-1A males (MST=93 hrs) died an average of 49 hrs (35%) earlier than recipient X1 strain males (MST=142 hrs). Even more dramatic, X1.B-1A females (MST=76 hrs) died an average of 62 hrs (45%) earlier than females from its background X1 strain (MST=138 hrs). In fact, not one X1.B-1A female (of 46 mice tested) survived to the MST of sensitive B strain females (99 hrs), with survival times ranging from 61–96 hrs (Figure 5). Accordingly, we now consider the X1.B-1A congenic line as a hypersensitive strain. Subcongenic lines for the X1.B-1A interval are in progress to capture and narrow the region to at least that identified by the B.X1-1.303 line (*i.e.*, 59–69.8 Mbp). For X1.B-1B, which carries *Shali5* – the putative male-specific QTL, males trended towards being significantly more resistant than X1 males (164 hrs *vs.* 142 hrs; *p*<0.10), whereas female X1.B-1B mice did not differ from X1 females (131 hrs *vs.* 138 hrs; *p*=0.59). These data correlated with earlier QTL analysis results and are consistent with *Shali5* containing one or more male-specific effector genes; however, additional mice are needed to more reliably validate these early findings. Our attempts to increase the sample size and to refine the *Shali5* region have been unsuccessful in multiple lines, due to extremely poor survival of pups containing B alleles for *Shali5*, without B alleles for *Shali1*.

The B.X1-1 consomic line carries homozygous X1 alleles for Chr 1, including *Shali1* and probably *Shali5* (MUGA SNP analysis showed a loss of the distal ∼3 Mbp of Chr 1). Males did not differ from B strain control males for any of the B.X1-1 consomic and congenic lines. B.X1-1 consomic females, which carry both *Shali1* and *Shali5*, also did not differ from B strain females (*p*=0.0076; compared to the Bonferroni corrected p-value of *p*<0.00132), but certainly trended towards the expected resistance. However, females for B.X1-1A (*p*=1.8×10^−4^), and B.X1-1AA (*p*=6.1×10^−4^) congenic lines differed significantly from B strain females. Both of these congenic lines carry *Shali1*, but not *Shali5*, supporting the resistance effect of *Shali1* X1 alleles. The B.X1-1.303 congenic line carries the segment around the microsatellite marker with the highest LOD score for *Shali1*. Again, B.X1-1.303 males did not differ from B strain controls. B.X1-1.303 females, however, were significantly more resistant than control B females (*p*=5×10^−7^), thereby potentially reducing the *Shali1* interval to ∼10.8 Mbp. As seen with the other B.X1 substituted Chr 1 lines, the MSTs for B.X1-1.303 males and females were similar. Since control B males were considerably more resistant than B females, these congenic lines suggest a possible female-specific resistance effect for *Shali1* X1 alleles (Figure 3).

#### *Chr 4: Shali2* (Figure 4)

The X1.B-4A congenic line, which does not include any part of the *Shali2* interval, showed an unpredicted increase in sensitivity for males (119 hrs *vs.* 142 hrs), and females (104 hrs *vs.* 138 hrs), although these did not reach significance due to high variability and low sample size. A similar result was found for the X1.B-4BA subcongenic line. For X1.B-4B, although just a dozen mice were generated and exposed to hyperoxia, results gave good indication that the X1.B-4B line contained one or more significant resistance loci (though only males were resistant in this small subset). The subcongenic line X1.B-4BB demonstrated a marked increase in MST for both males (*p*=2.1×10^−6^) and females (*p*=1×10^−6^). The X1.B-4C congenic was similar to X1.B-4B in B substituted alleles, except that it lost the distal ∼15 Mbp of Chr 4. Because the X1.B-4C line was considerably more fecund (frequent litters, often with 7–11 pups) than all other X1.B-Chr 4 lines to date, we initially tested this line more often. Like X1.B-4BB mice, X1.B-4C males were highly resistant compared to X1 males (*p*=2.7×10^−4^). But unlike the X1.B-4BB line, X1.B-4C females showed no survival time difference compared to X1 control females (n=34, *p*=0.98), suggesting potential female-specific susceptibility loci in the substituted regions differing between X1.B-4C and X1.B-4BB (*e.g.*, 83.5–114.5 Mbp or >141.4 Mbp) (Figure 4). In contrast to the X1.B Chr 4 lines, the reciprocal B.X1-4 consomic and congenic lines demonstrated no significant change in MSTs for males or females, when compared to the sensitive B strain controls. Because the B.X1-4BA and B.X1-4C congenic lines contain crossovers within the *Shali2* interval, their “no effect” outcome can potentially be combined with the crossovers in the X1.B-4 lines that did demonstrate an effect to help refine the *Shali2* segment. Subcongenic lines for the *Shali2* region are in progress.

### Penetrance of the high sensitivity and high resistance traits in consomic and congenic lines

Penetrance refers to the frequency in which individuals carrying a specific gene express the correlating trait. In this case, penetrance of the resistance trait was designated as a survival time >153 hrs, as defined previously [24]. Penetrance of high sensitivity was taken as the proportion of mice with a survival time less than the sex-specific MST of the sensitive B strain (*i.e.*, females <99 hrs and males <114 hrs). Table 1 gives the percent penetrance (sensitivity or resistance) for the relevant X1.B-1 and X1.B-4 congenic lines showing notable changes in MST, along with the percent penetrance of increased sensitivity and resistance for the B and X1 progenitor strains.

**Table 1:** Percent penetrance of sensitivity or resistance to hyperoxia-induced acute lung injury mortality.

	% Penetrance of Sensitivity		% Penetrance of Resistance	
Line	Males	Females	Males	Females
*Control Lines*				
B (C57BL/6J)	48	47	0	0
X1 (129X1/SvJ)	16	20	35	31
*Congenic Lines*				
X1.B-1A	88	100		
X1.B-1B			60	0
X1.B-4A	42	60		
X1.B-4BB			90	90
X1.B-4C			51	24

Of all sensitive B strain mice tested, none of either sex reached the threshold survival time for designation as “resistant”. However, 39/81 males and 32/68 females met the threshold for high sensitivity, resulting in 48% and 47% penetrance of the sensitivity trait in B strain males and females, respectively. For the X1 strain, 22/62 males and 23/74 females were resistant, giving 35 and 31% penetrance, respectively, for the resistance trait. Total penetrance for resistance (both sexes) was 33%.

Penetrance of the X1.B congenic lines differed strikingly from their background X1 strain in both directions, depending on the *Shali* substitution. Although X1 mice are considered a resistant strain (*i.e.*, 33% resistant), 18% of X1 mice (10/62 males and 15/74 females) showed high sensitivity, with a survival time less than the sex-matched mean of the sensitive B strain (Table 1). Remarkably, even though the B substitution was on the resistant X1 strain background, the penetrance of HALI sensitivity for the X1.B-1A congenic line was 93% of total mice, with 100% of X1.B-1A females (46/46) and 88% of males (59/67) demonstrating high sensitivity. In fact, of all X1.B-1A mice tested (n=113), only a single X1.B-1A male lived past the MST of the recipient X1 strain males (Figure 3). The X1.B-1B congenic line, which carries the male-specific *Shali5* region, had 60% penetrance of HALI resistance in males (6/10), but none of 8 females were resistant. Unexpectedly, the X1.B-4A congenic line, which contains no part of *Shali2*, demonstrated increased HALI sensitivity compared to X1 mice, with 5/12 males and 6/10 females showing highly sensitive survival times; only one of these 22 mice had a resistant phenotype. Although these data did not significantly differ from controls (due to small sample sizes relative to the reduced penetrance), the trends suggest further studies are needed for this putative susceptibility locus. The X1.B-4BB and X1.B-4C congenic lines both carry the *Shali2* interval. However, whereas the X1.B-4BB had 90% penetrance of resistance in males (19/21) and females (19/21), the X1.B-4C line showed increased penetrance of the resistance trait in males only (22/43 males vs. 8/33 females) when compared to the recipient X1 strain (Table 1).

## Discussion

Our previous QTL analysis of a large F_2_ population identified five putative QTLs affecting overall survival time to HALI [25]. Two of these QTLs, *Shali1* and *Shali2*, were validated as major effectors in a separate large backcross analysis [26]. Results of these recombinant populations generated from the B and X1 progenitor strains suggested that *Shali1* increased HALI resistance and MST when carried in homozygous X1 strain alleles, but *Shali2* increased resistance when homozygous for B strain alleles. The opposite scenario was also predicted, with homozygous B strain alleles for *Shali1* and homozygous X1 strain alleles for *Shali2* leading to increased sensitivity and earlier mortality in continuous >95% O_2_. Thus, the *Shali1* effect on HALI survival time directly correlated with the demonstrated outcome of the progenitors to a continuous hyperoxic exposure. To the contrary, the *Shali2* effect was masked within the progenitor strain background, as clearly exemplified by the fact that B strain mice carried the putative resistance alleles for *Shali2*, yet were HALI sensitive.

These large-effect QTLs with opposing allelic effects on HALI survival presented us with an intriguing opportunity to generate single congenic lines of mice that would synchronize these sensitivity or resistance QTLs in the same background strain and allow us to directly test *in vivo* our earlier *in silico* predictions. Because we were unsure whether either or both genetic backgrounds were important to support the predicted interactions, we generated reciprocal congenic lines for *Shali1* and for *Shali2* to coordinate the same effect QTLs in both the B and X1 backgrounds. This decision turned out to be critically important, since congenic strains for *Shali1* and *Shali2* generated on the B strain background showed much less effect compared to congenic lines on the recipient X1 strain. Since epistasis and other background effects cannot adequately be predicted for complex traits, these findings underscore the importance of generating reciprocal consomic and/or congenic strains to improve your chances of capturing the largest QTL effect(s).

This report presents the successful construction and subsequent testing of reciprocal congenic lines for the two major QTLs affecting HALI survival time. Most notably, we highlighted the dramatic and unpredicted phenotypic extremes demonstrated by the X1.B-1A (high penetrance of the sensitivity trait) and X1.B-4BB (high penetrance of the resistance trait) congenic strains. These lines were constructed to combine—within the same inbred strain—the reciprocal QTL effects of the two opposite-acting alleles for *Shali1* and *Shali2*, which originally derived from different chromosomes of the inbred X1 and B progenitor strains. In particular, the sensitive B alleles for *Shali1* were combined with the existing sensitive X1 alleles for *Shali2* in the X1 strain background, yielding the hypersensitive X1.B-1A congenic line. Based on our previous estimations for the *Shali1* effect [25], X1.B-1A mice were expected to have about a 15-hr increase in sensitivity from resistant X1 mice, predicting a MST of ∼115–120 hrs. Surprising, although on the resistant X1 strain background, the substitution of *Shali1* with B alleles made the X1.B-1A congenic line significantly more sensitive than the sensitive B strain, indicating that *Shali1* contained one or more key loci for overall HALI survival time. These results also strongly suggested that synchronizing the sensitivity alleles for *Shali1* and *Shali2* allowed direct interactions (either additively or synergistically) to further increase HALI sensitivity, and may signify important members of a similar pathway or process. As other sensitivity genes likely exist on the X1 strain background as well, the X1.B-1A line combines all resident sensitivity loci in the X1 strain with the *Shali1* sensitivity region transferred in from the B strain. In this case, the *Shali1* locus must contain at least one major-effect gene, which has the capacity to fully reverse the HALI phenotype of the resistant X1 strain. Therefore, the X1.B-1A data clearly validated the predicted sensitivity effect from *Shali1*-derived B strain alleles.

In the complementary strategy, resistant B alleles for *Shali2* were introgressed onto the X1 strain background, which already contained the resistant X1 alleles for *Shali1*. Together, the resistant allelic combination led to a large increase in HALI MST. In general, with the exception of a single female mouse with a survival time of 301 hrs (Figure 5), individual mice of X1.B-4BB did not live longer than the most resistant X1 strain mice (*i.e.*, 10–15% lived >200 hrs). Rather, because more than 90% of X1.B-4BB mice survived to the resistance threshold (and most survived significantly longer), the large increase in penetrance of the resistance trait can directly explain the 66-hr increase in MST of X1.B-4BB mice compared to the X1 strain. Again, effects larger than those predicted by the QTL analysis suggested an additive or potentially synergistic action for these two loci and/or other resistant loci present in the X1 strain background. In total, the resistance alleles for *Shali2* (B alleles) combined with alleles for *Shali1* and other existing resistance X1 alleles in the X1 strain background, almost totally eliminated the reduced penetrance of the resistance trait seen in the inbred X1 strain. Therefore, although the *Shali2* effect was masked in the inbred B and X1 strain genomes, data from the X1.B-4BB congenic line confirmed the predicted resistance effect of B-derived *Shali2* alleles.

Given the sizes of the chromosomal regions remaining in these congenic lines, it is premature to identify and speculate on potential positional candidate genes. Most congenics in this report contain extremely large regions of transferred DNA. Even the smallest region, which is currently in the B.X1-1.303 congenic strain, is more than 10 Mbp and contains hundreds of genes. Since the ∼10 Mbp region of B.X1-1.303 has not yet been captured on the more robust X1 strain background, we still consider this refined interval tentative. And, because it is not uncommon to see a single QTL dissect further into multiple, closely linked QTLs that often show opposing phenotypic effects [29], [31], [32], [35]–[39], further resolution of these regions and additional complementary strategies are needed before a highly informative candidate gene list can be generated. Because of their high penetrance, RNA-Seq analysis of the X1.B-1A and X1.B-4BB congenic lines will be used to help identify putative positional candidate genes for further study.

Besides this report, major QTLs on different chromosomes with counteracting effects within the same background strain have been published for other complex traits, although these findings are very rarely reported for rodents [40], [41]. The difficulties to detect such gene-gene interactions are likely to be an inherent shortcoming of the QTL analysis mapping strategy, suggesting that this will remain a significant challenge in complex trait analysis. For example, when two or more major genes of a complex trait have opposing effects, their interaction can partially or fully hide the overall measured effect, thereby leading to an inability to detect the individual QTLs or to a reduced capacity to determine their separate contributions. As described previously for lung cancer susceptibility [42], such complicated gene-gene interactions are likely to be commonplace in complex traits and contribute greatly to the heritable portion of phenotypic variation [43], [44]. Interestingly, the MST difference between the sensitive B (105 hrs) and resistant X1 (133 hrs) inbred strains was only about 1.26-fold. Traditional recommendations of using two polar-responding inbred strains for QTL analysis argues against using our model for QTL analysis. Although adhering to this recommendation will improve the odds of tracking and identifying QTLs, our data demonstrate that a large differential trait response between progenitors is not an absolute requirement. Given that the complexity of a trait is usually not known, many competing factors such as multiple genes, opposing effects, sex effects, gene-gene interactions and incomplete penetrance, can distort the overall measure differently on different background strains. For this reason, our established reciprocal congenic mouse models, which have captured significant gene-gene interactions for HALI susceptibility, provide us with unique and potentially powerful capacity to identify the critical genes for differential HALI susceptibility and to further characterize the complex interactions of these, and potentially other relevant background strain loci.

Additional findings with these reciprocal lines suggest other potential opportunities for follow-on breeding studies, either by creating specific informative multi-QTL congenics or by using other available congenic resources. First, the X1.B-1B line, which carries the proximal half of Chr 1, including the male-specific QTL *Shali5*, showed an increase in HALI resistance for males. As seen for *Shali2*, the B alleles were associated with increased resistance, although only males in the case of *Shali5*. These results suggest that a double congenic line containing the X1.B-4BB and X1.B-1B regions may show additional resistance for males by establishing a putative gene-gene interaction. Second, the X1.B-4A line carries the proximal region of Chr 4 derived from the B strain, an area not previously associated with a change in HALI survival time. However, the X1.B-4A mice demonstrated increased HALI sensitivity in females, suggesting a possible new sex-specific sensitivity QTL when homozygous for the B allele. Double congenics with the X1.B-1A and X1.B-4A lines could help validate this new QTL and their possible gene interactions for added sensitivity. Third, although the B.S congenics for both *Shali1* and *Shali2* often had little or no effects, select lines were statistically significant and, therefore, may provide potentially revealing information. For example, the substituted region that is associated with increased sensitivity in X1.B-1A mice is ∼102 Mbp (per MUGA SNP analysis). However, when combined with data from the B.X1-1.303 congenic line, the *Shali1* region can be tentatively refined to a 10.8-Mbp interval surrounding *D1Mit303*, from 59 to 69.8 Mbp (Figure 2). MUGA SNP analysis of B.X1-1C, which potentially carries an informative recombination in the *Shali1* interval, could further narrow the critical region. Because the X1 background shows more effect, this refined region can now be targeted for recombinants in the X1.B-1A line to validate this prediction, narrow the region of interest and allow selection of positional candidate genes. Thus, although the B strain background appears to be less informative for determining the *Shali* effects, their data in combination with the reciprocal lines can help narrow the important intervals. Fourth, because most knockout mice are often generated from 129-derived stem cells placed on the B strain background, our mouse model may allow us to take advantage of the knockout lines available for the genes that map to these QTL intervals to refine *Shali1* and S*hali2*, a strategy first proposed by Bolivar *et al.* [45]. This strategy would be most relevant for the B.X1-1.303 congenic line, which has demonstrated a significant effect on the B strain background.

In summary, these studies present the first description of congenic lines that validated two major QTLs on different chromosomes with opposite allelic effects for a complex trait in the same background inbred strain. These congenic lines further demonstrated large changes in the respective control strain phenotypes, which are indicative of these lines capturing and/or creating significant gene-gene interactions. The reciprocal congenic lines for *Shali1* and *Shali2* represent unique allelic mouse models to identify the quantitative trait genes for these QTLs and to critically assess the bidirectional epistatic interactions between these major-effect loci.
